# Multiple Tissues Transcriptome of Zig-Zag Eel (*Mastacembelus armatus*) with Different Growth Rates

**DOI:** 10.3390/ani14020248

**Published:** 2024-01-12

**Authors:** Jinlin Yang, Baoyue Lu, Zhide Yu, Linan Zhang, Yiman Chen, Zihui Chen, Chong Han, Hu Shu

**Affiliations:** School of Life Sciences, Guangzhou University, Guangzhou 510006, China; yangjinlin4739@163.com (J.Y.); lby12530@163.com (B.L.); 15902002970@163.com (Z.Y.); 18258972329@163.com (L.Z.); 1914130031@e.gzhu.edu.cn (Y.C.); 18320318247@163.com (Z.C.)

**Keywords:** *Mastacembelus armatus*, growth traits, transcriptome analysis, differentially expressed genes

## Abstract

**Simple Summary:**

In this study, transcriptome sequencing was first performed on the brain, liver, and muscle tissues of 3-month-old *M. armatus* individuals with different growth rates. A number of growth-related genes and biological pathways that related to skeletal muscle tissue development and insulin-like growth factor binding were screened. Our results suggest that *IGFBP-1A*, *IGFBP-1B*, *GH*, and *GHR* play an important role in the early developmental stage of *M. armatus*. These results contribute to providing a reference for further studies on the molecular mechanism of growth and development in teleost fish.

**Abstract:**

In order to explore the main regulatory genes and related pathways of growth traits, transcriptome sequencing was first performed on the brain, liver, and muscle tissues of 3-month-old *M. armatus* with different growth rates. By comparative transcriptome analysis of fast-growing and slow-growing groups of *M. armatus*, a total of 2887 DEGs were screened, of which 59 up-regulated genes and 105 down-regulated genes were detected in the brain, 146 up-regulated genes and 202 down-regulated genes were detected in the liver, and 529 up-regulated genes and 1846 down-regulated genes were detected in muscle, including insulin-like growth factor binding protein 1a (*IGFBP1A*), insulin-like growth factor binding protein 1b (*IGFBP1B*), myosin, light chain 1 (*MYL1*), and myoglobin (*MB*). Through Gene Ontology (GO) and the Kyoto Encyclopedia of Genes and Genomes (KEGG) enrichment analysis, we identified a total of 288 significantly enriched GO entries and 68 significantly enriched KEGG pathways related to growth, such as skeletal muscle tissue development, insulin-like growth factor binding, and the mitotic cell cycle. These key genes and signaling pathways may play a key role in regulating the growth of *M. armatus*. Digging into the regulatory mechanisms of these key genes will provide a theoretical basis for further exploration of the molecular mechanisms related to the growth and development of *M. armatus*, and help to breed new varieties of *M. armatus* with rapid growth.

## 1. Introduction

Fish growth refers to the physiological process in which the nutrients in the food are digested and absorbed into their own substances and continuously accumulated in the individual, which is mainly affected by factors such as genes, the environment, and nutrition. In the same fish population, some individuals grow faster than others. Studies have shown that some individuals in the same Atlantic sturgeon (*Acipenser oxyrinchus*) population have great differences in growth performance, especially in body length and weight [1]. In the same growing environment, the growth rate of different individuals in the same fish population [2] or different groups in the same fish population [3] will have obvious differences, which is the result of the genetic performance of individual fish and their long-term adaptation to the growing environment. In the same fish population, the growth rate is mainly regulated by the expression of related genes, the endocrine system, and other factors [4]. The growth rate of the same kind of fish is mainly regulated by external factors such as nutrition, breeding density, and temperature [5]. In the artificial farming process, the faster the growth rate of fish, the shorter the time required to reach the market specifications, which can greatly reduce the cost of time, labor, and so on, and further improve the economic benefits of artificial fish farming. Therefore, growth traits are often regarded as one of the most important economic traits to measure fish in artificial culture. With the continuous development of the aquaculture industry, people pay more and more attention to growth traits of fish, and related studies on growth traits are increasing.

Transcriptome RNA sequencing (RNA-Seq) technology is mainly used to study gene function or gene structure at the transcriptional level, reveal the biological process of gene expression in a specific growth stage or under a specific growth environment, and analyze the differentially expressed genes (DEGs) of individuals. The development of transcriptome sequencing technology has laid a technical foundation for studying the differential expression of genes in specific tissues and cells in a certain growth environment or growth stage [6]. At present, transcriptome analysis has been widely applied to the growth regulation of species. Sun et al. [3] revealed the relevant mechanisms of fish growth regulation by comparing the transcriptome between the offspring and parents of hybrid grouper, providing a reference for a deeper understanding of the molecular mechanism and regulatory pathway of the growth advantage of hybrid offspring. Similar studies have also been applied to several fish such as black porgy (*Acanthopagrus schlegelii*) [2], black carp (*Mylopharyngodon piceus*) [7], and bighead carp (*Hypophthalmichthys nobilis*) [8]. In order to reveal the main regulatory genes and pathways associated with the growth traits of black porgy, Lin et al. [2] conducted transcriptome sequencing analysis on the mixed samples of the brain, liver, and muscle tissue of 4-month-old black porgy with different growth rates, and concluded that the genes myosin heavy chain 4 (*MYH4*), insulin-like growth factor 1 (*IGF-1*), insulin-like growth factor 2 (*IGF-2*), myosin light chain 3-like (*Myl3*), myosin 6-like (*MYH6*), and myoglobin (*MB*) may have a negative impact on the growth of black porgy. At the same time, the important roles of the PPAR signaling pathway and GH-GHR-IGFs neuroendocrine pathway in the regulation of growth and development of black porgy were also revealed. Zhang et al. [7] conducted transcriptome analysis on muscle and liver tissues of the same batch of black carp with different growth rates, and found that epidermal growth factor (*EGF*) and vascular endothelial growth factor (*VEGF*) were significantly up-regulated in muscle, suggesting that these genes might be related to the growth rate of black carp. Fu et al. [8] used the RNA-Seq technology to further explore the regulatory role of the hypothalamus–pituitary–liver axis in the growth of bighead carp. They successfully identified three DEGs that were within the growth-related QTL related to the function of growth traits, and 164 lncRNAs specifically expressed in the liver and 749 lncRNAs specifically expressed in the hypothalamic–pituitary gland, which provided a large amount of basic data for revealing the genes associated with fish growth and their molecular mechanisms.

Zig-zag eel (*M. armatus*) belongs to the order Symbranchiformes, family Mastacembelidae, and genus *Mastacembelus*. The meat of *M. armatus* is tender and delicious with high protein content and unsaturated fatty acids, which makes it have very high research and development prospects [9]. However, up to now, there are few reports on the growth of *M. armatus*. Only Zhong et al. [10,11] cloned and expressed the growth hormone gene and myostatin (*MSTN*) gene of *M. armatus*. Thus, there is a serious lack of analytical studies on the growth and development of *M. armatus*. This study mainly focuses on *M. armatus* with different growth rates, and first analyzes the molecular genetic mechanism of growth and development in *M. armatus* with the help of RNA-Seq technology. The results will provide theoretical support for the genetic improvement of growth traits and molecular breeding of *M. armatus*.

## 2. Materials and Methods

### 2.1. Fish Maintenance and Specification Measurement

**Figure 1:**
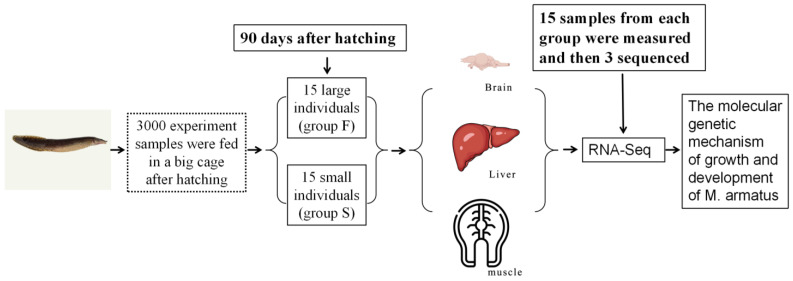
Experimental flow chart.

The *M. armatus* used in this study were from the same batch. The breeding experiment (July–October 2022) was carried out in Guangdong Lianyi Aquatic Science and Technology Development Co., Ltd. in Kaiping, China. The same batch of *M. armatus* were fed twice a day (8:00, 16:00) in a cage with the length, width, and height of 5 m, 2 m, and 3 m, and the feeding amount was 3% of the body weight. The average water temperature during the breeding period was 30 °C, the highest water temperature was 33.5 °C, and the lowest water temperature was 28 °C. All other conditions were controlled within the suitable range for the breeding of *M. armatus*. A total of 15 large individuals (group F) and 15 small individuals (group S) were selected from the whole population of *M. armatus* at 90 days after hatching (dah). We also sampled and measured their body length and weight respectively, and 3 individuals with the largest body weight and 3 individuals with the smallest body weight were selected as the study objects for transcriptome analysis. After being anesthetized with MS-222, the brain, liver, and muscle tissues were collected, frozen with liquid nitrogen, and stored in a −80 °C refrigerator for later use (Figure 1). All animal handling procedures and experimental protocols were approved by the Experimental Animal Ethics Committee of the Guangzhou University of China (No. URBBB220506).

### 2.2. RNA Extraction, Library Construction, and Transcriptome Sequencing RNA

Firstly, the total RNA of different tissue samples was extracted by Total RNA Extraction Reagent (Vazyme, Nanjing, China), and the extracted RNA was detected with a NanoDrop 2000 spectrophotometer (Thermo Fisher Scientific, Waltham, MA, America) and 1.5% TAE agarose gel electrophoresis. After the RNA samples were qualified, eukaryotic mRNA was enriched with magnetic beads with Oligo (dT). Subsequently, mRNA was broken into short fragments in fragmentation buffer. A cDNA strand was synthesized with six-base random hexamers based on an mRNA template. Then the buffer, dNTPS, DNA polymerase l, and RNase H were added to synthesize the double-stranded cDNA and the double-stranded cDNA was purified by AMPure XP beads. The purified double-stranded cDNA was first end-repaired, A-tailed, and connected to sequencing joints and then fragment size selection was carried out with AMPure XP beads. Finally, PCR was performed and the PCR products were purified by AMPure XP beads to obtain the final library. The libraries that passed the quality inspection were sequenced by the Illumina Hiseq 2500 high-throughput sequencing platform for PE150 sequencing.

### 2.3. Filtering of Clean Reads and Alignment Clean Reads

After obtaining the sequencing data of the sample, we used fastp (v.0.19.7, https://github.com/OpenGene/fastp) (accessed on 8 March 2023) [12] to further filter the original sequencing readings to remove low-quality data and ensure the confidence of the subsequent analysis results. Bowtie2 (v.2.33, https://github.com/BenLangmead/bowtie2) (accessed on 8 March 2023) [13] software was used to compare the sequencing data after quality control with the ribosome sequences in NCBI and Rfam, remove the matched sequences, and obtain the filtered data for subsequent analysis. Then HISAT2 (v.2.1.0, https://github.com/infphilo/hisat2) (accessed on 8 March 2023) [14] was used to compare the data after ribosome removal with the reference whole genome [15] to obtain the result file in BAM (Binary Alignment/Map) format. Finally, BAM file and GTF (Gene transfer format) annotation information were used to judge the quality of this sequencing.

### 2.4. DEGs and Enrichment Analysis

We used DESeq2 (v.1.28.1, https://www.bioconductor.org/packages/release/bioc/html/DESeq2.html) (accessed on 8 March 2023) [16] for gene differential expression analysis among samples, FPKM (Fragments Per Kilobase Per Million) was used to calculate gene expression levels, and DEGs were screened according to FDR (false discovery rate) ≤ 0.05 and /log2FC/ ≥ 1. After obtaining DEGs, the GO database was used to classify annotated genes and gene products in cell components, biological processes, and molecular functions, and reveal the functions of a specific group of genes with similar expression patterns. With DEGs as foreground genes and all expressed genes as background genes, clusterProfiler (v.3.4.4, http://www.bioconductor.org/packages/release/bioc/html/clusterprofiler.html) (accessed on 8 March 2023) [17,18] software was used for functional enrichment analysis of GO and KEGG. The next step was to transfer the analysis results to the cloud platform of Guangzhou Gidio Biotechnology Co., Ltd. in Guangzhou, China (https://www.omicshare.com/tools/) (accessed on 8 August 2023) in order to generate a circular chart.

### 2.5. Principal Component Analysis

Principal component analysis (PCA) in RNA-Seq was mainly used to analyze the relationship between samples. On the premise of ensuring that the information contained in the original data is retained as much as possible, the expression levels of thousands of genes contained in different tissue samples were reduced to a number of principal components for comparative analysis between samples. It reveals the structural relationship hidden behind the original data [19]. According to the values of each sample in the first principal component (PC1) and the second principal component (PC2), a two-dimensional coordinate plot was made. If the gene expressions of the samples were more similar, the distance between the points reflected in the PCA map was less, indicating a stronger correlation between the samples.

### 2.6. Real-Time Quantitative Polymerase Chain Reaction (RT-qPCR) Validation

To validate the transcriptome data of this study, we randomly selected 16 DEGs and analyzed their expression levels by RT-qPCR (Table 1). The reverse transcription of cDNA was performed according to the instructions of HiScript II Q RT SuperMix for RT-qPCR (+gDNA wiper) (Vazyme, China). The reverse transcription process was as follows: removal of genomic DNA at 42 °C for 2 min; 55 °C for 15 min; 85 °C for 5 s.

RT-qPCR was performed on the Roche 480 real-time PCR system according to the ChamQ SYBR qPCR Master Mix (Vazyme, China) kit instructions, using β-actin gene as the internal reference. The relative expression of selected genes was calculated by the 2^−∆∆Ct^ [20] method.

### 2.7. Statistical Analysis

SPSS statistics 26.0 software (SPSS Inc., Chicago, IL, USA) was used for statistical analysis. All the data were analyzed with a *t*-test. The resulting data were visually analyzed using GraphPad Prism 9 (GraphPad Software, San Diego, CA, USA). Differences were considered statistically significant at *p* < 0.05.

## 3. Results

### 3.1. Analysis of Growth Data

**Figure 2:**
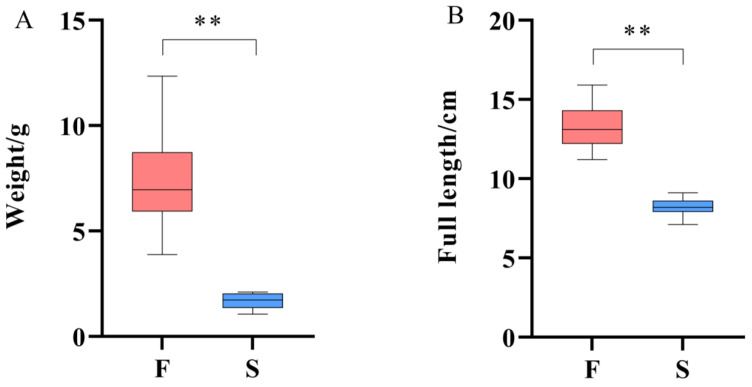
Weight and full length statistics of individuals with different growth rates. F: group F, S: group S, (**A**) is the analysis of significant differences in weight, (**B**) is the analysis of significant differences in full length, ** *p*-value below 0.01.

A total of 15 large individuals (group F) and 15 small individuals (group S) were selected from the whole population of *M. armatus* at 90 dah. Furthermore, we sampled and measured their body length and weight, respectively. The results showed that there were significant differences in body length and body weight between group F and group S (Figure 2). The average body weight of group F was 7.47 g, the maximum body weight was 12.35 g, and the minimum body weight was 3.88 g. The average full length of group F was 13.36 cm, the maximum full length was 15.90 cm, and the minimum full length was 11.20 cm. The average body weight of group S was 1.69 g, the maximum body weight was 2.11 g, and the minimum body weight was 1.05 g. The average full length of group S was 8.25 cm, the maximum full length was 9.10 cm, and the minimum full length was 7.10 cm.

### 3.2. Transcriptome Sequencing Data, Quality Processing, and Principal Component Analysis

Before the bioinformatics analysis, to ensure the quality of the sequencing data, we first used fastp software (v.0.19.7) to perform quality control on the original sequencing data and remove low-quality reads. After filtering out low-quality reads, the average total number of bases produced in brain tissue (SB), liver tissue (SL), and muscle tissue (SM) of the slow-growing group was 7.96 G, 6.85 G, and 7.13 G, respectively. In the fast-growing group, the average base total produced by brain tissue (FB) was 5.57 G, liver tissue (FL) was 6.10 G, and muscle tissue (FM) was 6.12 G. The average values of Q20 and Q30 were 98.93% and 95.74%, respectively. The GC content was 46.38~50.94%. The rates of clean bases of all samples were in the range of 89.03% to 90.69% (Table 2).

A total of 801,512,980 clean reads were obtained, after removing reads mapped to the rRNA database. The mapping rate of eighteen samples was 74.22–87.08% when the clean reads were aligned with the genome of *M. armatus* (Table 3).

**Figure 3:**
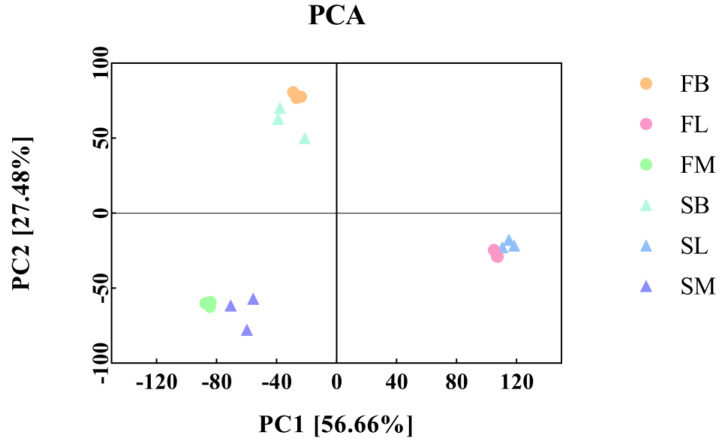
Sample principal component analysis (PCA). FB: Brain tissue from the sample individuals in group F. FL: Liver tissue from the sample individuals in group F. FM: Muscle tissue from the sample individuals in group F. SB: Brain tissue from the sample individuals in group S. SL: Liver tissue from the sample individuals in group S. SM: Muscle tissue from the sample individuals in group S.

Based on the gene expression information among different samples, we conducted principal component analysis (PCA). The PC1 coordinate represented the first principal component, and the contribution rate of the first principal component to the sample difference was 64.12%. The PC2 coordinate represented the second principal component, which contributed 30.69% to sample differences (Figure 3). The overall distribution and sample composition of *M. armatus* with different growth rates were different, and the samples in the group had good repeatability, which met the experimental requirements.

### 3.3. Screening and Verification of DEGs

**Figure 4:**
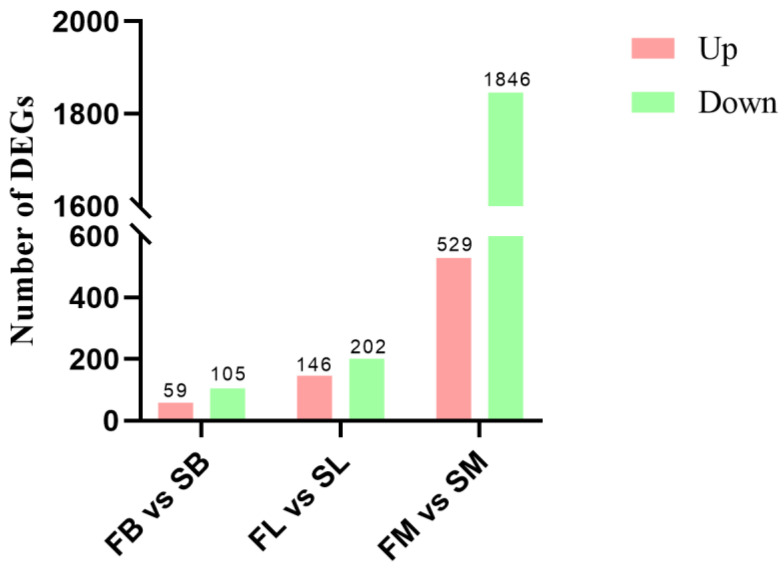
Statistics of differentially expressed genes.

**Figure 5:**
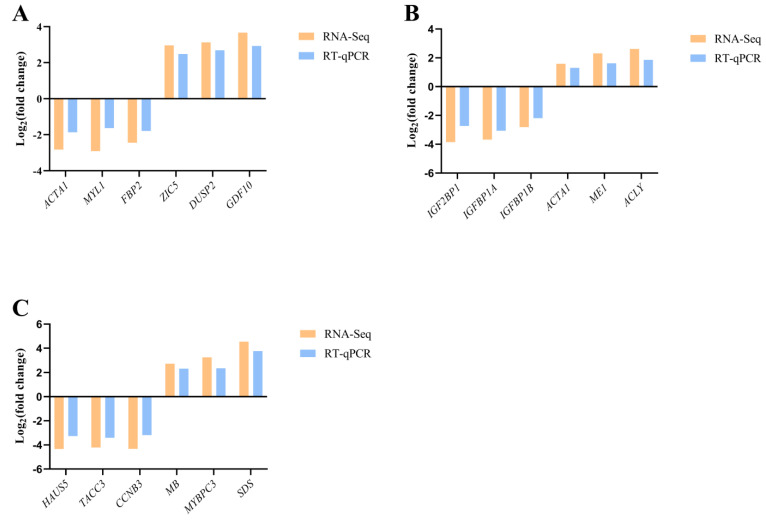
Gene expression patterns of RNA-Seq and RT-qPCR. (**A**) The results of RT-qPCR identification of DEGs in brain tissue. (**B**) The results of RT-qPCR identification of DEGs in liver tissue. (**C**) The RT-qPCR identification results of DEGs in muscle tissue. *ACTA1*: actin alpha 1, skeletal muscle b. *MYL1*: myosin, light chain 1, alkali; skeletal, fast. *ZIC5*: zic family member 5 (odd-paired homolog, *Drosophila*). *FBP2*: fructose-1,6-bisphosphatase 2. *GDF10*: growth differentiation factor 10b. *DUSP2*: dual specificity phosphatase 2. *IGF2BP1*: insulin-like growth factor 2 mRNA binding protein 1. *IGFBP1A*: insulin-like growth factor binding protein 1a. *IGFBP1B*: insulin-like growth factor binding protein 1b. *ME1*: malic enzyme 1, NADP(+)-dependent, cytosolic. *ACLYA*: ATP citrate lyase a. *MYBPC3*: myosin binding protein C3. *SDS*: serine dehydratase-like. *HAUS5*: HAUS augmin-like complex, subunit 5. *TACC3*: transforming, acidic coiled-coil containing protein 3. *CCNB3*: cyclin B3.

The transcriptome data of group F and group S were compared and analyzed. A total of 2887 significant DEGs (FDR ≤ 0.05, |log2FC| ≥ 1) were found in group F as controls, including 734 up-regulated genes and 2153 down-regulated genes (Figure 4). In order to further verify the accuracy of transcriptome data, six DEGs were randomly selected from transcriptional data of the brain, liver, and muscles, respectively, for RT-qPCR validation (Table 1). The results showed that the RT-qPCR identification results and regulatory trends (increase or decrease) of 16 randomly selected DEGs were basically consistent with the transcriptomic data (Figure 5), proving the reliability of transcriptomic sequencing results.

### 3.4. GO and KEGG Enrichment Analysis

**Figure 6:**
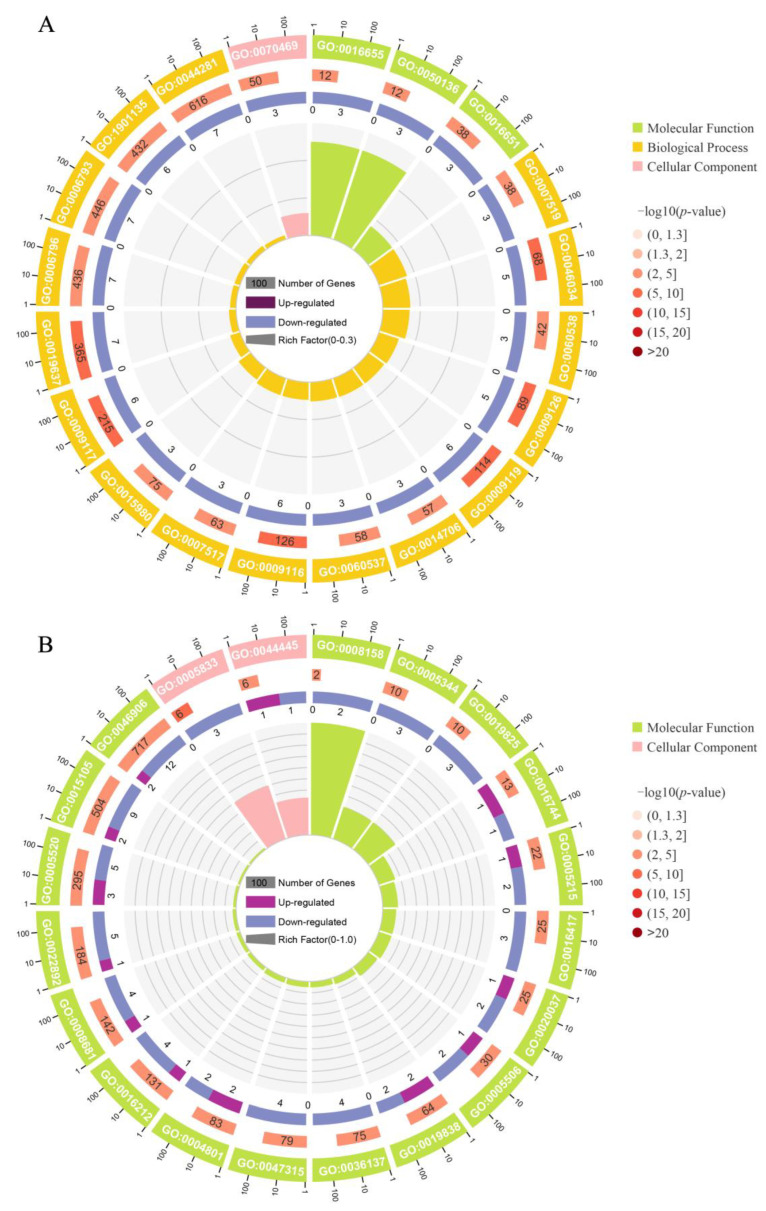

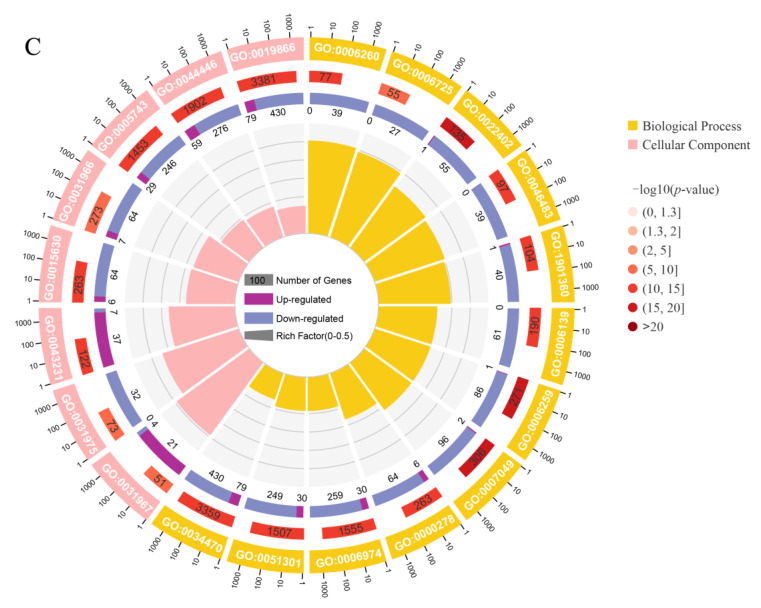
GO functional annotation analysis of DEGs in brain (**A**), liver (**B**), and muscle (**C**). Circle diagram (from the outside in, the first circle: enrich the top 25 GO terms, different colors represent different categories. Outside the circle is the coordinate scale of the number of genes. The second circle: the number of GO terms in the background gene and the *p*-values. A longer bar means more genes, and a redder color means a smaller number. The third circle: the bar chart of up- and down-regulated genes, dark purple represents the up-regulated gene ratio, light purple represents the down-regulated gene ratio. The specific values are shown below. The fourth circle: RichFactor values for each GO term, with background auxiliary lines representing 0.1 in each grid).

GO enrichment analysis and functional annotation were carried out on 2887 DEGs of the F and S groups. We found that the DEGs of FB vs. SB, FL vs. SL, and FM vs. SM were enriched into 207, 199, and 595 GO items, respectively (*p* ≤ 0.05). These GO items included skeletal muscle tissue development and metabolic process in FB vs. SB, insulin-like growth factor binding in FL vs. SL, cell cycle process, DNA replication, mitotic cell cycle, and mitotic cell cycle process in FM vs. SM. Among them, there were 288 significantly enriched GO items (Q value ≤ 0.01) (Table S1), which included skeletal muscle tissue development, insulin-like growth factor binding, mitotic cell cycle, etc. The top 20 of the three different tissue GO enrichment terms were shown in Figure 6, and the GO numbers in the figure were provided in the Supplementary Materials (Tables S2–S4).

**Figure 7:**
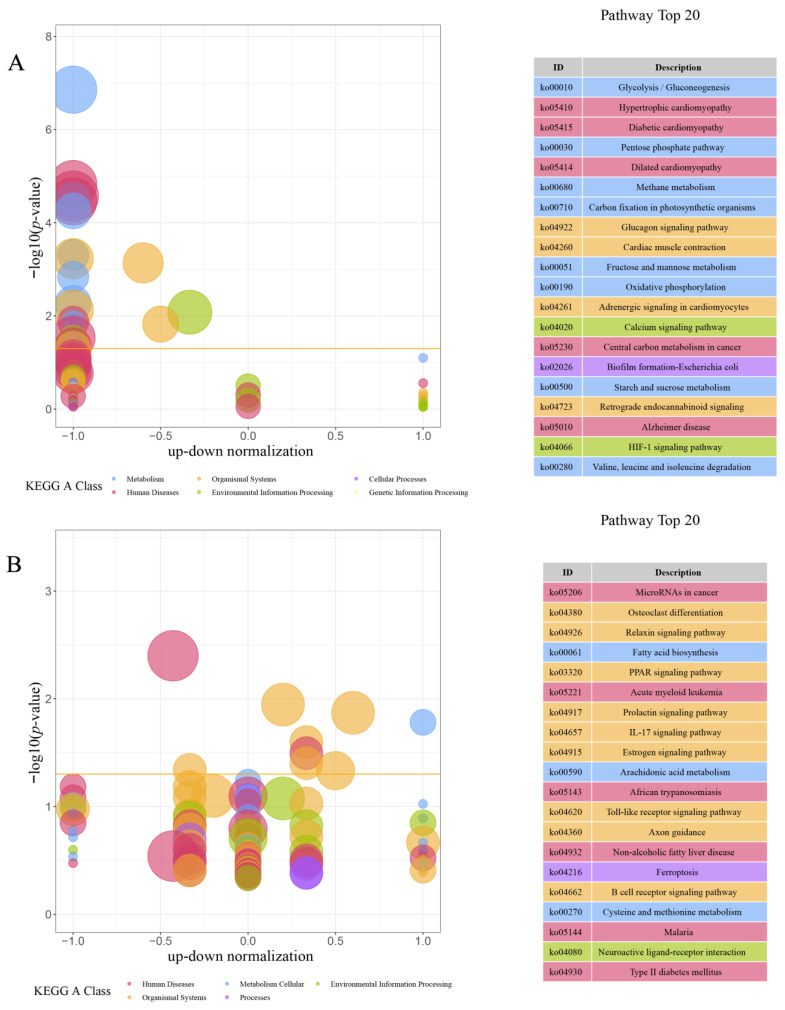

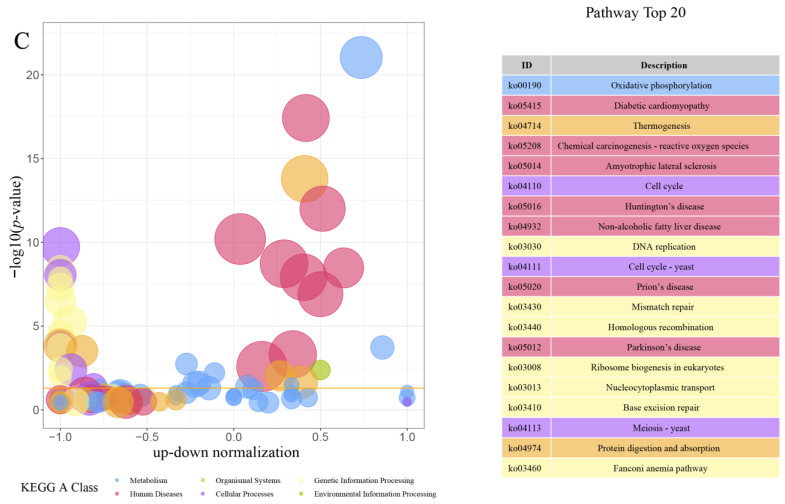
KEGG enrichment analysis of DEGs in brain (**A**), liver (**B**), and muscle (**C**). The horizontal axis of the bubble diagram is the up–down normalization coefficient; the vertical axis is −log10pvalue; different colors indicate different functional classifications; the orange threshold is *p*-value = 0.05; the size of the bubble indicates the number of genes enriched in the current term (pathway) (i.e., up + down); the table on the right shows the 20 pathways with the smallest *p*-value.

In order to explore the major signaling pathways related to the growth rate of *M. armatus*, KEGG functional enrichment analysis was performed on 2887 DEGs. A total of 22 significant related pathways were screened out of 89 enrichment pathways in FB vs. SB, such as glycolysis/gluconeogenesis, pentose phosphate pathway, glucagon signaling pathway, etc. (Figure 7A). Among 188 enrichment pathways of FL vs. SL, 9 significant related pathways were screened, such as fatty acid biosynthesis and the PPAR signaling pathway (Figure 7B). Among 367 enrichment pathways of FM vs. SM, 37 significant related pathways were screened, such as DNA replication, protein digestion and absorption, the citrate cycle (TCA (tricarboxylic acid) cycle), P53 signaling pathway, etc. (Figure 7C). The top 20 KEGG enrichment pathways were shown in Figure 7, and the details of the classes and paths were added to the Supplementary Material (Tables S5–S7).

### 3.5. Identification of Differentially Expressed Growth Genes

In the DEGs of *M. armatus* with different growth rates in this study, based on the GO and KEGG annotation, we found that 18 growth-related genes were up-regulated or down-regulated in group F. The down-regulated genes in brain tissue were *ACTA1B*, *MYLPFA*, *MYL1*, *TNNT3A*, *MYLZ3*, *LDB3B*, *ACTN3B*, and *SYNPO2LA*, while the up-regulated gene was *ZIC5* (Table 4). Down-regulated genes in liver tissues included *IGF2BP1*, *IGFBP1A*, *IGFBP1B*, *ITGA7*, and *IRS2B*, while up-regulated genes included *ACLY* and *MYL9B* (Table 5). Down-regulated genes in muscle tissue included *CDKN1A*, *IGFBP1B*, *IGF2BP1*, and *IGF2BP3*, while up-regulated genes included *MYOM3* and *MB* (Table 6).

## 4. Discussion

In recent years, owing to the rapid development of transcriptome sequencing technology, researchers have been able to conduct in-depth studies on gene expression, gene function, signaling pathways, and other aspects [21]. At present, transcriptome analysis technology has been widely used in related studies on developmental biology [22,23], environmental stress [24], adaptive evolution [25], immunology [26], and growth rate [27].

This study found that under the same breeding conditions, there were significant differences in the growth rate of the same batch of *M. armatus* seedlings. Transcriptome sequencing was performed on brain, liver, and muscle tissue samples of 3-month-old *M. armatus* with different growth rates by high-throughput sequencing technology. A total of 2887 DEGs were screened, including *IGFBP1B*, *ACLY*, *ACTA1*, *IGFBP1A*, *MYL1*, *MB*, and other key genes involved in regulating body growth.

The effects of growth hormone on cell growth are mainly mediated by the growth hormone/somatomedin (*GH/IGF*) growth-axis [28]. It is a major activator of the growth hormone/somatomedin (*GH/IGF*) growth-axis, stimulating *IGF-I* secretion in liver or other target tissues [29,30]. The original auxin hypothesis put forward that *GH* mainly promoted the growth and differentiation of cells by stimulating the liver to secrete *IGF-I*, and then *IGF-I* is circulated to the target organ through the blood circulation [31]. Interestingly, the role of growth hormone in promoting individual growth seems to be due to the direct effect of growth hormone on target tissues, rather than the circulating *IGF-I* mediated by liver production [32]. Studies have shown that growth hormone can not only stimulate the secretion of *IGF-I* in liver tissues to increase the level of *IGF-I* in plasma, but it can also affect the *IGF-I* produced by target tissues to act in an autocrine or paracrine way [33]. Meanwhile, based on relevant experimental data of gene knockout mice, circulating *IGF-I* is mainly derived from *IGF-I* produced by the liver, and the influence of circulating *IGF-I* on growth may be little or even nonexistent, while locally produced *IGF-I* in target tissues may be the reason why *IGF-I* promotes growth [34]. In an mRNA localization experiment of the *IGF-I* and *IGF-I* receptor in gilthead sea bream (*Sparus aurata*), Funkenstein et al. [35] found that in the early growth and development of fish larvae, *IGF-I* might stimulate cell proliferation in target tissues by paracrine or autocrine means, while the liver did not play an important role in *IGF-I* production. When we subsequently verified the expression of growth-related genes in *M. armatus* by RT-qPCR, it was found that the expression of *GH* in group F was significantly higher than that in group S in brain tissue, and there was no difference in the expression of *IGF-I* between group F and group S in liver tissue, while the expression of *IGF-I* in the muscle of group F was significantly higher than that in group S. It seemed that the growth-promoting effect of *GH* might be directly exerted on the growth tissues in the early developmental stage of *M. armatus*, rather than mediated by liver secretion of *IGF-I*. Meanwhile, the expression of *GH* and *GHR* in the muscle tissue of group F was significantly higher than that in group S, indicating that, like gilthead sea bream [32,35], in the early developmental stage of *M. armatus*, *GH* acted directly by binding to the *GH* receptor (*GHR*) in the target tissue (e.g., muscle tissue), and induced the target tissue to secrete *IGF-I* in an autocrine/paracrine manner to participate in the regulation of tissue growth and differentiation. Notably, Silva et al. [36] created double transgenic zebrafish (*Danio rerio*) and the utilization of the highly expressed *GHR* in zebrafish muscle tissue reduced the negative effects of overexpressed *GH* on reproduction. Creating double transgenic *M. armatus* overexpressing both the growth hormone (*GH*) and its receptor (*GHR*) may not only effectively reduce the negative effects of growth hormone, but also further achieve maximal growth and minimal side effects and significantly improve the growth rate of *M. armatus* in commercial aquaculture settings.

Under the stimulation of growth hormone (*GH*), the liver secretes insulin-like growth factor (*IGF*)-*I* [37]. *IGFBP-1* can bind to *IGF* and regulate the role of *IGF* in the growth and development of somatic cells [38]. Due to its high affinity for *IGF-I*, *IGFBP-1* can prevent *IGF-I* from interacting with other receptors and inhibit the effect of *IGF-I* after binding with *IGF-I* [39]. Since the *IGFBP-1* of fish has no nuclear localization sequence, *IGFBP-1* plays a role mainly by regulating the action of *IGF-1* [40]. Studies have shown that circulating *IGFBP-1* can inhibit the interaction between the *IGF-1* and *IGF* receptor on pituitary cells [40], and *IGFBP-1A* can inhibit the action of *IGF-1* [41]. Because *IGFBP-1B* has a higher dissociation rate, *IGFBP-1B* has a much lower affinity for *IGF-I* and *-II* than *IGFBP-1A*, and both *IGFBP-1A* and *IGFBP-1B* inhibit the *IGF-1*-induced cell proliferation process during embryonic development in zebrafish (*Danio rerio*). Overexpression of *IGFBP-1B* can significantly reduce the growth and development rate of embryos [38]. It is concluded that *IGFBP-1A* and *IGFBP-1B* may play an important role in the growth regulation of *M. armatus* seedlings, and the expression levels of *IGFBP-1A* and *IGFBP-1B* are significantly increased in slow-growing individuals. In this experiment, *IGFBP-1A* and *IGFBP-1B* were significantly down-regulated in fast-growing *M. armatus* seedlings, which was consistent with the above research results.

Myoglobin (*MB*) is an oxygen-binding protein, which can effectively promote oxygen diffusion in the muscle and is an efficient oxygen carrier [42]. It has been thought to be a protein only found in muscle cells [43], but later studies have shown that *MB* is not only expressed in the muscles of the Tibetan Plateau endemic schizothoracine fish (*Schizopygopsis pylzovi*), but also in the liver, muscle, kidney, brain, eyes, and skin [44]. *MB*, which is rich in bioavailable iron [45], can also clear active oxygen in vivo [46], store oxygen and promote intracellular oxygen diffusion [43], and regulate the concentration of nitric oxide in the sarcoplasm [42]. Transcriptome analysis of mixed tissues of black porgy (*Acanthopagrus schlegelii*) [2] showed that the expression of *MB* was significantly down-regulated in the slow-growing group. At the same time, some studies have shown that while the cultured Pacific bluefin tuna (*Thunnus orientalis*) grows, its *MB* content also increases continuously (*p* ≤ 0.05) [47]. Through transcriptome studies on grass carp (*Ctenopharyngodon idella*) with different growth rates, it was found that the expression of *MB* in fast-growing individuals was significantly higher than that in slow-growing individuals [48]. In this study, *MB* was significantly upregulated in the muscle tissue of fast-growing *M. armatus* individuals, similar to the results of the above study.

## 5. Conclusions

Transcriptome sequencing and bioinformatics analysis were performed on the brain, liver, and muscle tissues of *M. armatus* with different growth rates. Based on the results of this study, DEGs *IGFBP-1A*, *IGFBP-1B*, and *MB* were selected for analysis, and the expression of *GH*, *IGF-Ⅰ* and *GHR* of growth-related genes were also verified by RT-qPCR. The expression levels of *IGFBP-1A* and *IGFBP-1B* were significantly increased in the liver tissue of slow-growing *M. armatus* individuals, which might have a negative impact on the growth rate of *M. armatus*. *GH*, *GHR*, and *MB* were significantly up-regulated in fast-growing *M. armatus* individuals. From these results, we can infer that, in the early developmental stage of *M. armatus*, *GH* may act directly through binding to *GH* receptors (*GHR*) in muscle tissues. The results of this study provide a reference for further research on the molecular mechanism of growth and development in teleost fish.

## Figures and Tables

**Table 1:** RT-qPCR primers of differentially expressed genes.

Gene Name	Primer Sequence (5′→3′)	Melting Temperature/°C
*ACTA1*	F: GCACCACACCTTCTACAA	49.5
	R: AGCATCCAGCACAATACC	49.7
*MYL1*	F: GACCTGTCCTCCATCAAG	49
	R: AGCCATTCTCATCCTCCT	49.3
*Z* *IC5*	F: GCACGAACTGGTCAATCA	50.5
	R: TGTGAGTCCTCTTGTGAATC	50.4
*FBP2*	F: GCCATCTACAAGCGGTTAT	50.5
	R: GGTCCAGCATGAAGAAGTT	50.5
*GDF10*	F: TCCAGCCAACCTACCATAT	50.2
	R: CCTTCTTCATCGTCCTCTC	49.9
*DUSP2*	F: GGTGGCAGATGAGAACAG	50.2
	R: AGTCAATGAAGGCGATGG	49.8
*IGF2BP1*	F: CAGACGCAGAGCAAGATT	50.2
	R: AAGTTGTTGTGAGCCAGAA	50.2
*IGFBP1* *A*	F: AGAGCCTGAGATGGAGAAC	51.1
	R: CCAGAGACGACTCACACT	50.7
*IGFBP1* *B*	F: CAGCACTGGACACGATAG	50.1
	R: TGACTTCTTGATGACACTCT	49.3
*M* *E1*	F: TCACGAGAAGGAGAGGTT	49.3
	R: GGAAGATGTAGGCATTGTTC	49.8
*ACLYA*	F: GGTATGTCCAACGAACTCA	50
	R: TCTGTGCCTCCAATCTCT	49.6
*MYBPC3*	F: GCCTCCTGTCCTGATTGTGA	55.0
	R: AGCCATAGATTCTCCGCCATT	55.1
*SDS*	F: TGCTGAATGGAGTGGTGGAG	55.3
	R: CCTGGTCTGTGACTACTTCTGA	54.9
*HAUS5*	F: ATCCATAACAGAGCCACAAGTC	54.2
	R: ACATCCAGAGCAGCCAGTT	54.2
*TACC3*	F: ACTGAGAACCTGCCGAACAA	55.1
	R: AAGGACTCCATTGCTGCTGTA	55.2
*CCNB3*	F: TCTCAGTGTCTGTCAGGTTGT	54.4
	R: GAGTTCTCAGCGTCAATGTCA	54.3

**Table 2:** Data quality control results of each sample.

Sample	Clean Paired Reads	Clean Bases (G)	Q20 (%)	Q30 (%)	GC Content (%)	Clean Data Ratio (%)
SB-1	52,431,126	7.63	98.89	95.63	46.97	89.03
SB-2	52,766,502	7.7	98.94	95.81	47.4	89.36
SB-3	58,687,688	8.55	98.86	95.53	47.29	89.32
SL-1	43,273,446	6.32	98.96	95.81	48.69	90.69
SL-2	50,526,274	7.37	99.02	96.03	49.28	90.26
SL-3	46,867,612	6.85	99.01	96.01	49.01	90.15
SM-1	45,096,312	6.61	98.97	95.86	50.44	90.3
SM-2	53,343,670	7.8	98.99	95.98	49.89	90.07
SM-3	47,723,246	6.99	99.06	96.19	50.41	90.64
FB-1	37,071,366	5.45	98.77	95.22	46.61	90.16
FB-2	36,020,462	5.3	98.74	95.14	46.38	89.86
FB-3	40,536,446	5.97	98.75	95.18	46.91	89.6
FL-1	40,133,186	5.87	98.89	95.62	48.61	89.05
FL-2	41,425,186	6.04	99.06	96.17	49.01	89.94
FL-3	43,314,210	6.4	98.81	95.34	48.48	89.45
FM-1	39,622,272	5.82	98.97	95.89	50.94	90.27
FM-2	40,047,894	5.85	99.02	96.03	50.78	89.99
FM-3	45,607,112	6.68	98.95	95.83	50.73	90.12

Sample: Sample name; Clean Paired Reads: The total number of reads with junctions and low-quality bases were filtered out; Clean Bases: The total number of filtered bases, that is, the number of clean reads multiplied by the length; Q20 (%): The percentage of bases with a correct base recognition rate of more than 99%; Q30 (%): The percentage of bases with a correct base recognition rate of more than 99.9%; GC Content (%): The number of G + C as a percentage of the total number of bases; Clean Data Ratio (%): Rate of clean bases.

**Table 3:** Alignment results of the reference genome.

Sample	Total Reads	Paired Mapped Reads (%)	Unpaired Mapped Reads (%)	Unmapped Reads (%)	Total Mapped Reads (%)
SB-1	51,517,494	38,344,392 (74.43%)	3,161,940 (6.14%)	10,011,162 (19.43%)	41,506,332 (80.57%)
SB-2	51,613,212	36,682,906 (71.07%)	3,175,401 (6.15%)	11,754,905 (22.77%)	39,858,307 (77.23%)
SB-3	57,178,028	40,208,324 (70.32%)	3,507,018 (6.13%)	13,462,686 (23.55%)	43,715,342 (76.45%)
SL-1	42,519,140	30,457,448 (71.63%)	2,904,978 (6.83%)	9,156,714 (21.54%)	33,362,426 (78.46%)
SL-2	49,550,142	35,433,480 (71.51%)	3,105,560 (6.27%)	11,011,102 (22.22%)	38,539,040 (77.78%)
SL-3	45,301,398	31,087,930 (68.62%)	3,084,443 (6.81%)	11,129,025 (24.57%)	34,172,373 (75.43%)
SM-1	44,805,510	36,928,266 (82.42%)	2,044,978 (4.56%)	5,832,266 (13.02%)	38,973,244 (86.98%)
SM-2	53,099,480	40,588,834 (76.44%)	3,085,672 (5.81%)	9,424,974 (17.75%)	43,674,506 (82.25%)
SM-3	47,381,560	38,406,404 (81.06%)	2,163,296 (4.57%)	6,811,860 (14.38%)	40,569,700 (85.62%)
FB-1	36,626,502	25,861,926 (70.61%)	2,666,815 (7.28%)	8,097,761 (22.11%)	28,528,741 (77.89%)
FB-2	35,652,742	24,567,788 (68.91%)	2,849,139 (7.99%)	8,235,815 (23.10%)	27,416,927 (76.90%)
FB-3	39,921,844	28,220,434 (70.69%)	2,825,609 (7.08%)	8,875,801 (22.23%)	31,046,043 (77.77%)
FL-1	39,241,700	26,444,650 (67.39%)	2,679,001 (6.83%)	10,118,049 (25.78%)	29,123,651 (74.22%)
FL-2	40,425,420	27,880,806 (68.97%)	2,630,530 (6.51%)	9,914,084 (24.52%)	30,511,336 (75.48%)
FL-3	42,839,680	28,279,204 (66.01%)	3,870,241 (9.03%)	10,690,235 (24.95%)	32,149,445 (75.05%)
FM-1	38,794,238	32,069,580 (82.67%)	1,665,771 (4.29%)	5,058,887 (13.04%)	33,735,351 (86.96%)
FM-2	39,748,678	33,003,178 (83.03%)	1,608,890 (4.05%)	5,136,610 (12.92%)	34,612,068 (87.08%)
FM-3	45,296,212	37,355,274 (82.47%)	2,009,298 (4.44%)	5,931,640 (13.10%)	39,364,572 (86.90%)

Total Reads: The number of clean reads after rRNA filtering; Paired Mapped Reads: The number of clean reads that mapped to multiple sites of the reference genome; Unpaired Mapped Reads: The number of clean reads that mapped to a single site of the reference genome; Unmapped Reads: The number of clean reads that were unmapped to the reference genome; Total Mapped Reads: The number of clean reads that mapped to the reference genome.

**Table 4:** Differentially expressed growth genes in brain tissue.

ID	Log2FC (FDR < 0.05)	Gene	Description
113122805	−2.822629123	*ACTA1B*	Actin alpha 1, skeletal muscle b.
113141556	−2.92542818	*MYLPFA*	Myosin light chain, phosphorylatable, fast skeletal muscle a.
113123291	−2.913046983	*MYL1*	Myosin, light chain 1, alkali; skeletal, fast.
113132910	−3.131707241	*TNNT3A*	Troponin T type 3a (skeletal, fast).
113127528	−3.002337731	*MYLZ3*	Myosin, light polypeptide 3, skeletal muscle.
113135302	−2.965010599	*LDB3* *B*	LIM domain binding 3b.
113135777	−2.476991992	*ACTN3B*	Actinin alpha 3b.
113123100	2.966333471	*ZIC5*	Zic family member 5.
113132015	−2.568776028	*SYNPO2LA*	Synaptopodin 2-like a.

**Table 5:** Differentially expressed growth genes in liver tissue.

ID	Log2FC (FDR < 0.05)	Gene	Description
113141522	−3.854791781	*IGF2BP1*	Insulin-like growth factor 2 mRNA binding protein 1.
113139767	−3.682401004	*IGFBP1* *A*	Insulin-like growth factor binding protein 1a.
113134287	−2.810482959	*IGFBP1* *B*	Insulin-like growth factor binding protein 1b.
113130337	−2.274576177	*ITGA7*	Integrin, alpha 7.
113122938	−2.001084859	*IRS2B*	Insulin receptor substrate 2b
113141142	2.623058494	*ACLYA*	ATP citrate lyase a.
113130328	2.160598057	*MYL9B*	Myosin, light chain 9b, regulatory.

**Table 6:** Differentially expressed growth genes in muscle tissue.

ID	Log2FC (FDR < 0.05)	Gene	Description
113130167	−3.846745356	*CDKN1* *A*	Cyclin-dependent kinase inhibitor 1A.
113134287	−3.870604985	*IGFBP1* *B*	Insulin-like growth factor binding protein 1b.
113141522	−2.699824093	*IGF2BP1*	Insulin-like growth factor 2 mRNA binding protein 1.
113126273	−2.520499238	*IGF2BP3*	Insulin-like growth factor 2 mRNA binding protein 3.
113136432	2.379825043	*MYOM3*	Myomesin 3.
113128157	2.737550286	*MB*	Myoglobin.

## Data Availability

The raw data are available from the SRA (http://www.ncbi.nlm.nih.gov/sra/) (accessed on 17 November 2023) data repository (accession number: PRJNA1053665).

## Supplementary Materials

The following supporting information can be downloaded at: https://www.mdpi.com/article/10.3390/ani14020248/s1, Table S1: Significantly enriched GO terms; Table S2: The GO number of picture A in Figure 6; Table S3: The GO number of picture B in Figure 6; Table S4: The GO number of picture C in Figure 6; Table S5: The pathway of picture A in Figure 7; Table S6: The pathway of picture B in Figure 7; Table S7: The pathway of picture C in Figure 7.
